# Endovascular Stent Grafting for Aortic Arch Aneurysm in Aortoiliac Occlusive Disease following Aortic Arch Debranching and Aortobifemoral Reconstruction

**DOI:** 10.1155/2017/6568028

**Published:** 2017-03-16

**Authors:** Didem Melis Oztas, Cagla Canbay, Yilmaz Onal, Metin Onur Beyaz, Omer Ali Sayin, Mehmet Barburoglu, Mehmet Buget, Mesut Yornuk, Aziz Ari, Murat Ugurlucan, Bulent Acunas, Ufuk Alpagut, Enver Dayioglu

**Affiliations:** ^1^Department of Cardiovascular Surgery, Istanbul Medical Faculty, Istanbul University, Istanbul, Turkey; ^2^Department of Radiology, Istanbul Medical Faculty, Istanbul University, Istanbul, Turkey; ^3^Department of Anesthesia, Istanbul Medical Faculty, Istanbul University, Istanbul, Turkey; ^4^Istanbul Training and Research Hospital, Department of General Surgery, Istanbul, Turkey

## Abstract

Treatment of thoracic aortic aneurysms constitutes high mortality and morbidity rates despite improvements in surgery, anesthesia, and technology. Endovascular stent grafting may be an alternative therapy with lower risks when compared with conventional techniques. However, sometimes the branches of the aortic arch may require transport to the proximal segments prior to successful thoracic aortic endovascular stent grafting. Atherosclerosis is accounted among the etiology of both aneurysms and occlusive diseases that can coexist in the same patient. In these situations stent grafting may even be more complicated. In this report, we present the treatment of a 92-year-old patient with aortic arch aneurysm and proximal descending aortic aneurysm. For successful thoracic endovascular stent grafting, the patient needed an alternative route other than the native femoral and iliac arteries for the deployment of the stent graft. In addition, debranching of left carotid and subclavian arteries from the aortic arch was also required for successful exclusion of the thoracic aneurysm.

## 1. Introduction 

Current treatment protocols for aneurysms in descending aorta include conventional open surgery or endovascular techniques. However, mortality and morbidity rates are still high with open surgical methods [1]. In particular, the ratio increases when the pathology extends to the aortic arch. Conventional total aortic arch replacement for the treatment of aortic arch aneurysms requires deep hypothermia, cardiac arrest, extracorporeal circulation, and total circulatory arrest. The risk is somehow reduced with combination of endovascular and open surgical approaches [2]. In selected cases, hybrid arch repair eliminates circulatory arrest and hence reduces the mortality and morbidity rates [3].

In this report, we present the treatment of a patient with descending thoracic aortic aneurysm and aortic arch aneurysm together with aortoiliac occlusive disease.

## 2. Case Report 

**Figure 1:**
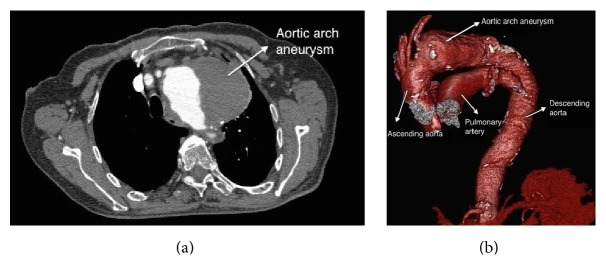
(a) Computed tomography (CT) angiography view of 10 cm diameter aneurysm which includes zone 2 area. (b) Computed tomography (CT) angiography view of 10 cm diameter aneurysm which includes zone 2 area.

A 92-year-old male patient was referred to our clinic with aortic arch aneurysm. The patient was hypertensive and active smoker. The computed tomography (CT) angiography revealed a 10 cm diameter aneurysm which included zone 2 area in thoracic aorta (Figures 1(a) and 1(b)). Thoracic endovascular stent grafting (TEVAR) was planned due to age and the comorbidity factors of the patient. Separation of the left common carotid and left subclavian arteries from the aortic arch (debranching) was required for safe treatment of the aneurysm.

## 3. Surgical Technique 

**Figure 2:**
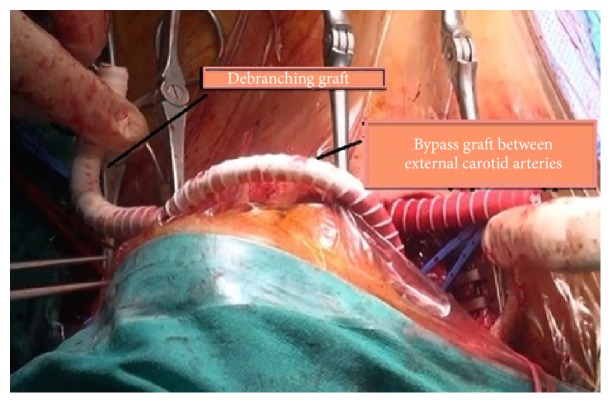
The bypass performed on the skin by 6 mm PTFE graft between both external carotid arteries is seen.

**Figure 3:**
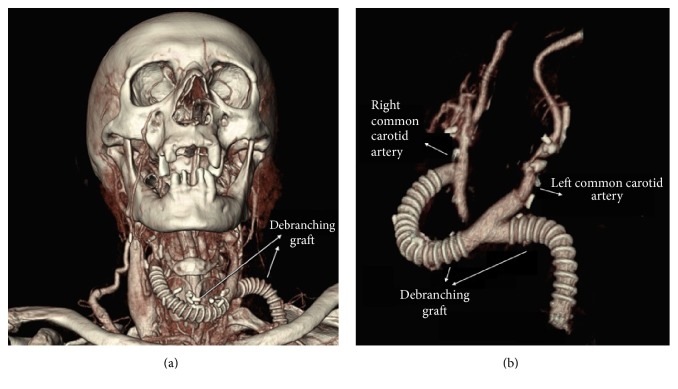
(a) Postoperative computed tomography (CT) angiography view of the debranching graft. (b) Debranching graft's proximal anastomosis was performed end to side to right common carotid artery; graft's carotid leg was anastomosed to left common carotid artery end to end; graft's left subclavian artery leg was anastomosed to the left subclavian artery end to side; and left subclavian artery was simply ligated proximally.

Aortic arch debranching was performed according to Ugurlucan's technique [4, 5]. The operation was performed with the left cervical and subclavian infiltration anesthesia and right cervical regional block. Standard incisions parallel to the bilateral sternocleidomastoid muscles were performed. The right and left common, internal and external carotid arteries were dissected. Left subclavian artery was prepared with left supraclavicular incision. Debranching Y bypass graft was prepared from an 8 mm ringed PTFE graft. Graft was passed through the tunnels to the desired regions. After heparinization carotid arteries were clamped consecutively; however, patient experienced neurologic changes in 15 seconds. A crossover bypass with a 6 mm PTFE graft was performed between bilateral external carotid arteries above the skin (Figure 2). Right common carotid artery was clamped. Proximal part of the debranching bypass graft was anastomosed end to side to the right common carotid artery. The left common carotid artery was clamped and transected. The proximal stump was sutured primarily. The carotid limb of the debranching graft was anastomosed end to end to the left common carotid artery. The crossover external carotid artery bypass between the external carotid arteries is no longer needed for cerebral protection after that step. It was simply clamped and separated from the external carotid arteries. The left subclavian artery was clamped and the left subclavian limb of the debranching graft was anastomosed end to side to the left subclavian artery (Figures 3(a) and 3(b)). Then the subclavian artery was simply ligated proximal to the left vertebral artery.

Deaeration of the grafts was performed carefully before removal of the arterial clamps. No cerebral ischemic period occurred during or after the procedure. Pulsatile flow in the internal carotid arteries was never interrupted [4].

## 4. Endovascular Stent Graft Implantation Technique

The stent grafting of aneurysm including zone 2 aortic arch was planned in elective conditions. He underwent endovascular grafting after 6 days of supra-aortic debranching operation. Right femoral incision was performed and common, deep, and superficial femoral arteries were dissected. The left femoral artery was cannulated for angiographic images. The stent grafting was attempted using the right femoral artery but failed due to iliac stenosis. After balloon angioplasty to right iliac artery, procedure was repeated but it failed again. Then, we decided to continue through the left femoral artery. The left femoral artery was dissected; however, again stent graft deployment could not be successful due to left iliac stenosis. We balloon-dilated the left iliac artery. Control angiography was performed because of sudden hypotension and we detected left iliac rupture. The balloon was inflated in infrarenal aorta. A stent graft (16 × 16 × 120 mm, Endurant Medtronic Endovascular, Santa Roja, CA, US) was implanted to the left common iliac artery and ruptured iliac artery was repaired. Stent graft deployment was attempted through the stent graft implanted into the left iliac artery but could not be successful again.

The patient had abdominal aortic aneurysm (infrarenal abdominal aorta diameter 4.3 cm) and biiliac stenosis. Hence, we planned aortobifemoral bypass (ABF) graft operation and stent graft implantation (hybrid procedure) through the bifurcated graft to the aortic arch even though both iliac arteries seemed to have enough lumen diameter for the deployment of the stent graft and after failure of multiple attempts and balloon dilatation and even iliac stent graft implantation.

## 5. Hybrid Procedures 

The patient underwent the aortobifemoral bypass graft operation after 7 days of failed TEVAR attempt. Median laparotomy was performed with general anesthesia. We performed aneurysm resection and ABF operation with a 22 × 11 mm Dacron graft (Vascutec, Terumo, Renfrewshire, Scotland, UK) for the treatment of infrarenal abdominal aortic aneurysm and biiliac stenosis. After the closure of the laparotomy incision, the patient was taken to the catheter laboratory.

Right brachial artery was cannulated for control angiograms. The left limb of Dacron ABF graft was incised. Endovascular stent graft (23 × 14 × 105 mm, Endurant Medtronic Endovascular, Santa Roja, CA, USA) was inserted into the proper position with the guidance of stiff wire and aortic arch aneurysm was successfully excluded. Left femoral graft was primarily repaired and femoral incisions were closed. The patient was transferred to the intensive care unit.

**Figure 4:**
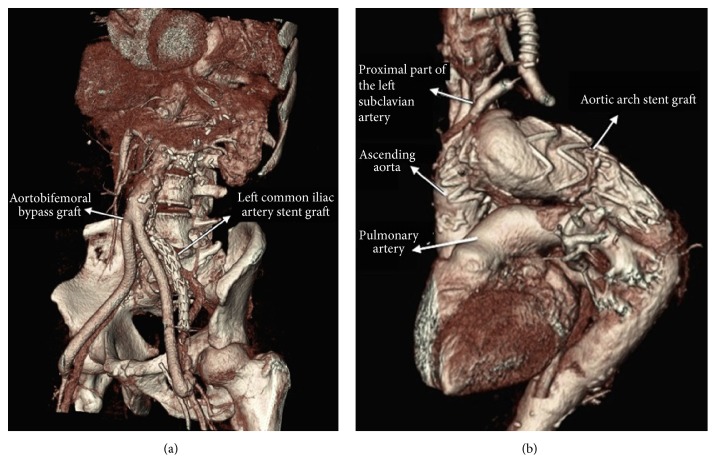
(a) Postoperative control CT angiography image of the stent graft which was applied to the left common iliac artery and the ABF. (b) Postoperative CT angiography view of the stent graft which was used in TEVAR procedure.

Postoperative control CT angiography revealed successful ABF and TEVAR procedures (Figures 4(a) and 4(b)). Early postoperative course was uneventful. Unfortunately, the patient died due to natural causes in the early term follow-up.

## 6. Discussion 

Aneurysms confined to the thoracic aorta are observed in 4.5 of every 100,000 people. They are generally asymptomatic and detected incidentally during radiologic imaging studies [5]. They can cause symptoms such as chest pain, back pain, dysphagia, dyspnea, hoarseness, and recurrent laryngeal nerve compression. Some complications such as aortic rupture and dissection may be life threatening.

The morbidity and mortality rates of conventional open surgical procedures are still high despite technologic and medical advances. Although the mortality of open surgical therapies in thoracic aortic aneurysms has been reported to be as low as 2-3% in the best series, patients with comorbidities and older age still have high mortality rates [6]. Stroke rate after surgery ranges between 2 and 18% [7]. Hence, there has always been a search for alternative treatment modalities for the treatment of thoracic aortic aneurysms in the medical era. Endovascular stent grafting evolved rapidly in recent years and proposed to be a promising alternative to open surgery which may decrease morbidity and mortality rates either alone or when performed in combination with open surgical procedures especially in patients with high risks [8, 9].

The complex anatomy of the aortic arch led physicians to divide it into zones in order to facilitate description of the aneurysms as well as treatment modalities [10]. Our patient had an aneurysm confined to zone 2. In addition, the complex anatomy of the aortic arch makes endovascular stent grafting challenging due to the technical difficulties of the design and implantation of the branched stent grafts. Hence, various techniques are developed to make these pathologies suitable for endovascular stent graft treatment. Debranching, elephant trunk operation, chimney techniques, and branched modular stent grafts are among these techniques. As in our patient, the distal aortic arch lesions, that is, zones 2 and 3 [10], may require coverage of the left carotid and subclavian artery orifices for a successful stent graft placement and exclusion of the aneurysm. It may be necessary to revascularize these arteries before a TEVAR, which will include the aortic arch [4, 11, 12]. Our group has invented a novel debranching procedure: cerebral protection with a crossover bypass between right and left external carotid arteries which provides pulsatile blood flow to the brain despite proximal arterial clamp on the consecutively clamped carotid arteries and therefore minimizes the neurologic complication rates [4, 5]. Revascularization of the left subclavian artery may not be necessary in many instances. Simple vascular plug occlusion with angiographic methods could also be an option to prevent endoleak rather than revascularization of the left arm and proximal subclavian artery ligation. However, bypass to the left subclavian artery was simple and easily performed intentionally, in a fashion to permit antegrade vertebral pulsatile flow, to prevent any cerebellar unexpected complications, not with a reason regarding spinal cord ischemia, as it is well known that risk of spinal cord ischemia is very low in patients receiving TEVAR less than 20 cm.

Femoral arteries are the most frequently used vessels for the deployment of the stent grafts during endovascular stent grafting procedures. The stent graft system is then passed through the iliac arteries to its final destination. Suitable vascular bed and sufficient vessel diameter are necessary for this procedure. In cases when the femoral arteries have insufficient calibers, the iliac arteries or aorta itself can directly be used for stent graft deployment.

Atherosclerosis is the common etiology of aneurysms and arterial occlusive diseases. Therefore, iliofemoral stenosis may coexist in patients with aneurysms. In these cases, for a successful endovascular therapy, reconstruction of the stenotic segment with prosthetic graft or stents may enable the deployment. Method has been defined as an alternative option in challenging cases [13, 14]. In our case, abdominal aortic aneurysm together with aortoiliac occlusive disease did not allow us to do endovascular stent grafting, although the measured diameters of the iliac arteries on preoperative CT and despite post-appropriate sized balloon angioplasty seemed feasible for the stent graft delivery system deployment. Due to the very old age of our patient, conventional surgical repair of the thoracic aortic aneurysm and abdominal aortic aneurysm with aortoiliac occlusive disease would be too much and was not preferred. This has to be accounted as the major limitation of our case. Rather, we preferred an additional hybrid approach to cervical debranching; that is, we performed surgical replacement of the abdominal aorta and iliac arteries with an ABF operation with a big size Dacron graft which facilitated endovascular stent grafting. As a result complete arterial reconstruction could be performed with various endovascular and hybrid surgical approaches in our patient, successfully.

## 7. Conclusion 

Endovascular interventions are used frequently to complement surgical treatment in high-risk patients. Hybrid treatment methods enable significant decrease in morbidity and mortality rates when compared with conventional techniques especially in patients with comorbidities and older age.

Aortic arch aneurysms may require debranching prior to TEVAR procedure. Cerebral protection is very important to prevent stroke. We provided continuous pulsatile flow in internal carotid arteries despite proximal clamping with temporary bypass between external carotid arteries in our case [Ugurlucan's cerebral protection technique during aortic arch debranching [4, 5]] for aortic arch debranching and successful exclusion of the aneurysm confined to the thoracic aorta. In addition, as in our case for the failing TEVAR procedures due to inappropriate sized arterial access, additional hybrid procedure can be performed to facilitate the TEVAR [14] and to treat accompanying compromising pathologies.

## Abbreviations

CT: Computed tomography
TEVAR: Thoracic endovascular stent grafting
ABF: Aortobifemoral bypass.

## References

[1] Kouchoukos N. T., Dougenis D. (1997). Surgery of the thoracic aorta. *New England Journal of Medicine*.

[2] Usui A., Ueda Y., Watanabe T. (2000). Clinical results of implantation of an endovascular covered stent-graft via midsternotomy for distal aortic arch aneurysm. *Cardiovascular Surgery*.

[3] Cochennec F., Tresson P., Cross J., Desgranges P., Allaire E., Becquemin J.-P. (2013). Hybrid repair of aortic arch dissections. *Journal of Vascular Surgery*.

[4] Ugurlucan M., Sayin O. A., Onalan M. A. (2015). Cerebral protection with a crossover external carotid artery bypass during arch debranching. *Annals of Thoracic Surgery*.

[5] Ugurlucan M., Alpagut U. (2015). Treatment solution by the readers: endovascular treatment of aortic arch aneurysms following aortic debranching. *Interactive Cardiovascular and Thoracic Surgery*.

[6] Duru S., Erdem M., Agca E., Kaplan T., Ardic S. (2013). Thoracic aortic aneurysm: a rare case report. *Turk Toraks Dergisi*.

[7] Köksal C., Özcan V., Sarikaya S., Meydan B., Zengin M., Numan F. (2004). Endovascular treatment of thoracic aortic aneurysms. *Anadolu Kardiyoloji Dergisi*.

[8] Moon M. C., Morales J. P., Greenberg R. K. (2007). The aortic arch and ascending aorta: are they within the endovascular realm?. *Seminars in Vascular Surgery*.

[9] Esposito G., Marullo A. G. M., Pennetta A. R. (2008). Hybrid treatment of thoracoabdominal aortic aneurysms with the use of a new prosthesis. *Annals of Thoracic Surgery*.

[10] Mitchell R. S., Ishimaru S., Ehrlich M. P. (2002). First International Summit on Thoracic Aortic Endografting: roundtable on thoracic aortic dissection as an indication for endografting. *Journal of Endovascular Therapy*.

[11] Starnes B. W., Tran N. T., McDonald J. M. (2007). Hybrid Approaches to Repair of Complex Aortic Aneurysmal Disease. *Surgical Clinics of North America*.

[12] Numan F., Gülşen F., Arbatli H., Cantaşdemir M., Solak S. (2011). Aort anevrizmalarının endovasküler tedavisinde yeni ufuklar. *Turkish Journal of Thoracic and Cardiovascular Surgery*.

[13] Ugurlucan M., Akyol Y., Guven K. (2007). Treatment of chronic type B aortic dissection by endovascular grafting in a previously CABG patient. Case report. *Acta Chirurgica Belgica*.

[14] Alpagut U., Ugurlucan M., Dayioglu E. (2007). Endovascular treatment of thoracic aortic pathologies in patients with aortoiliac occlusive disease. *The Heart Surgery Forum*.

